# Pupillometry to show stress release during equine sports massage therapy

**DOI:** 10.1038/s41598-023-47590-y

**Published:** 2023-11-27

**Authors:** Karen Nicola Wild, Stephan Skiba, Suvi Räsänen, Claus-Peter Richter

**Affiliations:** 1 Hands for Horses Sweden, Murkelvägen 78, 186 56 Vallentuna, Sweden; 2 SkImagine, Fatburs Brunnsgata 26 LGH 1403, 118 28 Stockholm, Sweden; 3 SR Häst- Och Ryttarutbildning, Vreta 1, 186 93 Vallentuna, Sweden; 4 Feinberg School of Medicine, Department of Otolaryngology, Northwestern University, 320 E. Superior Street, Searle 13-564, Chicago, IL 60611 USA; 5 Department of Communication Sciences and Disorders, Northwestern University, 70 Arts Circle Drive, Evanston, IL 60208 USA; 6 Department of Biomedical Engineering, Northwestern University, 2145 Sheridan Road, Tech E310, Evanston, IL 60201 USA; 7 The Hugh Knowles Center, Department of Communication Sciences and Disorders, Northwestern University, 70 Arts Circle Drive, Evanston, IL 60208 USA

**Keywords:** Behavioural methods, Biophysical methods, Biological techniques, Biomarkers, Diagnostic markers

## Abstract

Anecdotal reports state that wellness treatments for horses, such as massage therapy, relaxes the treated animal. Massage therapists and horse owners typically report an ”improvement” without verifying or quantifying the treatment results. This paper shows that the effect of wellness treatment and stress release can be measured with pupillometry. One of the horse’s pupils was photographed at the beginning and end of the treatment to determine the changes in the balance between the sympathetic and parasympathetic system activities. The owners assigned horses to two experimental groups: animals receiving a massage (N = 18) and horses standing with a person next to the horse for the time of a massage in the stable lane (N = 10). Six animals in the experimental group were excluded from the analysis because the pupils could not be traced. We opened the images of the pupil with Fiji (ImageJ) and used the elliptical selection tool to measure the pupils’ and iris’ areas. The ratio between the pupils’ aperture and the iris’ area was a normalized measure for pupil size. At the end of the experiment, we compared the normalized size of the pupils with a two-tailed paired t-test within groups and a two-tailed t-test between groups. For the experimental group, it was before and after the treatment, and for the control group, before and after the horse was placed in the stable lane. Comparisons between the experimental and control groups were made at the procedure's beginning and end. The treatment significantly decreased the normalized pupil area in the experimental group, on average, by a factor of 0.78 ± 0.15 (*P* = 0.042). For the horses in the control group, the pupil size increased, on average, by a factor of 1.14 ± 018. Changes were statistically not significant (*P* = 0.19). The initial pupil size of the horses in the experimental group was 1.88 times larger than that in the control group. After the treatment, the difference was reduced to a factor of 1.25. At the beginning of the experiment, the horses in the experimental group had, on average, larger pupil sizes than the horses in the control group, indicating that the horses in the experimental group were more stressed before the treatment than the control animals. The observed changes in pupil size in the experimental group likely resulted from enhanced parasympathetic and diminished sympathetic activity resulting from the treatment. Observed changes in pupil size agree with the anecdotal horse owner reports and the therapist’s treatment notes.

## Introduction

While veterinarians successfully treat various locomotor disorders in horses, the healing of the injury does not complete the animal's recovery. Additional rehabilitative measures seek to overcome persistent limitations in movement or strength. Methods include manual therapy, physical or mechanical agents, electrotherapy, hydrotherapy, and exercise. Most of the published work is anecdotal, including expert opinions, reports on single cases, or the outcomes of small cohort treatments^1–5^. The conclusions of a recent comprehensive review on equine rehabilitation^1^ summarize the published work by stating that *“there is a lack of randomized clinical trials using large samples that can help describe evidence related to the different approaches cited.”* The conclusion continues by stating that *“some studies present options and parameterizations that can be useful for equine clinical practice, but it is clear that more evidence is needed with regard to parameters for use and efficacy of different rehabilitation methods in horses.”* Without going into detail about each of the published works, we agree with the statements of Atalaia et al.^1^ and propose and evaluate pupillometry as a method demonstrating the effect of sports massage therapy for horses. One of the objectives of the treatment is to improve wellness and release stress, which can be caused, for example, by tense muscles and pain. We propose that determining the change in pupil size can demonstrate a shift in sympathetic to parasympathetic activity or stress release and may serve to quantify treatment success.

In mammals, two muscle groups control pupil size, the iris sphincter muscle, a smooth muscle, and the iris dilator muscle, composed of myoepithelial cells (contractile elements)^6–8^. The iris sphincter muscle connects to the iris's vascularized fibrovascular layer and contracts the pupil^8,9^. Parasympathetic nerve fibers originating from the Edinger-Westphal nucleus of the fifth cranial nerve innervate this muscle^10–12^. The sympathetic system, releasing noradrenalin during stress situations, innervates the antagonist, the dilatator pupillae, which increases the pupil’s diameter^9,13^.

Among many other factors, stress activates the sympathetic nervous system, enlarging pupil diameter by a measurable amount^14–16^. Hence, pupil size may serve as a quantitative measure of stress or stress release. Previous research supports this hypothesis, reporting increased pupil diameters for stress and decreasing during stress release^14–18^. A good correlation between blood cortisol level and pupil diameter exists in fish. An increased cortisol level, typically seen under stress, increases pupil diameter^19^.

Note that the pupil's primary function in the mammalian eye is to regulate luminance by changing the pupil's diameter^20–24^. Pupil diameter changes also adjust the eye's aperture to optimize visual acuity for different light levels^25,26^. Therefore, it is important to control the light exposure if pupil size is used to determine a change in the balance between sympathetic and parasympathetic activity.

We have used the connection between stress and pupil diameter to answer whether pupil diameter changes before and after a horse receives massage therapy. The decrease in pupil diameter after the treatment indicates decreased stress levels obtained by the procedure. Today, treatment outcome measures are anecdotal reports by horse owners describing the “improvement” of the animal after the treatment. Typically, the criteria to judge the changes in the horse are the animals' behavior during riding exercises. A questionnaire could be used to assess and quantify these changes by assigning a numeric value to each outcome measure. However, such evaluations are still indirect measures involving the horses’ owners and are subject to personal bias.

This study aims to present a quantitative measure verifying the effect of the treatment by a change in stress level identified by the animals' pupils' diameter. The outcomes are qualitatively compared to the verbally communicated treatment results by the therapist, including an evaluation of the horse before and after the treatment.

## Results

### Anecdotal horse owner reports and therapist notes

Before the treatment, the horse owners reported that their horses were stiff in their left or right-hand movements. The horses might have resisted during riding exercises such as canter or sideways movements and had less ability to bend or show flexibility in their movements. Often, the horse owners reported a decrease in the range of the animal’s motion and that the subjective riding experience was less harmonic.

The horse owners’ reports reflected the massage therapist’s findings during the animal’s first assessment. For example, if the horse owner reported problems during the left-hand canter, the massage therapist would notify tension in the involved muscle groups. During the palpation, the horse showed hyper-reflexibility in the tense muscles, as determined by muscle fasciculations during palpation.

After the treatment, the tense muscle areas before the treatment were less tense, or the tension resolved. The horse showed either reduced or no reaction to palpation after the treatment. The reports were not detailed enough for a quantitative assessment.

### The horses

Table 1 shows the results from twelve of the eighteen horses in the experimental group, and Table 2 shows the ten horses in the control group. Six horses in the experimental group were removed from the analysis because the pupils could not be traced. Among the horses in the experimental group (Table 1), 6 (50%) were geldings (males), and 6 (50%) were mares (females). The animals’ age was similar for both genders. It ranged from 5 to 30 years for mares and geldings combined. The median age was 10 years, and the average age ± one standard deviation was 12.1 ± 6.4 years (Table 1). The mares’ ages ranged from 8 to 17 years, with a median of 9.5 years and an average age ± one standard deviation of 10.3 ± 3.4 years (Table 1). The age of the geldings ranged from 5 to 30 years, with a median of 12.5 years and an average age ± one standard deviation of 13.8 ± 8.5 years (Table 1). Horses in the control group are listed in Table 2. Six (60%) were geldings (males), and 4 (40%) were mares (females). The age of the animals was similar for both genders. For mares and geldings combined, the animals’ ages ranged from 10 to 20 years, with a median of 11 years and an average age ± one standard deviation of 12.0 ± 3.0 years (Table 2). The mares’ ages ranged from 10 to 13 years, with a median of 12 years and an average age ± one standard deviation of 11.8 ± 1.5 years (Table 2). The age of the geldings ranged from 10 to 20 years, with a median of 12.2 years and an average age ± one standard deviation of 11.0 ± 3.9 years (Table 2).

**Table 1:** Summarized results of the experimental group before and after treatment.

Identifier	Before	After	Ratio area2/area1	Horse information			
	Normalized area1	Normalized area2		Sex	Age	Discipline	Stable
H3	0.44	0.26	0.58	Gelding	30	Retired/leisure	1
H5	0.40	0.28	0.72	Mare	8	Eventing	2
H6	0.34	0.26	0.79	Gelding	13	Dressage	3
H7	0.47	0.35	0.75	Gelding	10	Leisure	3
H8	0.46	0.32	0.69	Mare	9	Jumping	3
H9	0.19	0.14	0.73	Gelding	12	Jumping/dressage	3
H11	0.20	0.13	0.64	Mare	10	Dressage/leisure	4
H13	0.24	0.21	0.89	Gelding	5	Dressage/leisure	4
H14	0.41	0.29	0.71	Mare	8	Dressage/leisure	4
H15	0.43	0.34	0.78	Mare	17	Dressage/leisure	5
H17	0.27	0.24	0.89	Mare	10	Dressage/leisure	3
H18	0.19	0.22	1.17	Gelding	13	Jumping	1
avg	0.34	0.25	0.78				
stdev	0.11	0.07	0.15				

The identifier provides information on the horses in the study. The ratio between the number of pixels counted in the traced pupil and the number counted in the traced iris is the normalized area 1 before treatment and area 2 after treatment. The most right column shows the relative decrease in area size after treatment. The bottom two rows show the average values and one standard deviation, respectively.

**Table 2:** Summarized results of the control group.

Identifier	Before	After	Ratio area2/area1	Horse information			
	Normalized area1	Normalized area2		Sex	Age	Discipline	Stable
C1	0.14	0.18	1.25	Gelding	11	dressage	5
C2	0.15	0.16	1.06	Gelding	20	dressage	5
C3	0.17	0.21	1.24	Mare	13	dressage	5
C4	0.21	0.22	1.03	Gelding	10	dressage	5
C5	0.22	0.26	1.16	Mare	11	dressage	5
C6	0.21	0.20	0.96	Gelding	11	dressage	5
C7	0.19	0.28	1.43	Mare	10	dressage	5
C8	0.20	0.16	0.80	Gelding	11	dressage	5
C9	0.11	0.14	1.27	Mare	13	dressage	5
C10	0.14	0.18	1.22	Gelding	10	dressage	5
avg	0.18	0.20	1.14				
stdev	0.04	0.04	0.18				

The identifier provides information on the horses in the study. The ratio between the number of pixels counted in the traced pupil and the number counted in the traced iris is the normalized area 1 at the beginning and the normalized area 2 after a defined time. The most right column shows the relative change in area size. The bottom two rows show the average values and one standard deviation, respectively.

Age differences were tested for significance with a t-test comparing two mean values of two distributions (IGOR Pro8 statistical package). Differences were not statistically significant for all males and females in the study (*P* = 0.32), males and females in the experimental group (*P* = 0.38), and males and females in the control group (*P* = 0.82). Similarly, age differences between the geldings in the experimental and the control groups were not statistically significant (*P* = 0.67). Age differences between the mares in the experimental and the control groups were not statistically significant (P = 0.40).

### Pupil area assessment

To control the brightness of the ambient light, the horses were treated inside the stable, where the stables’ lighting system determined the luminous flux. While the light intensity expressed in lumens was not measured for each horse, it was kept constant for each animal. Selecting this approach allowed determining the change in pupil size for each horse but introduced variability in the absolute values between animals. As described in Methods, for each horse, the measured and normalized pupil size was the area of the pupil’s aperture after the horse was led into the stable lane and had at least 15 min to acclimate to the surrounding environment. Similarly, the pupil size was determined after the session ended and before the horse was led back to its box.

#### Treatment reduced the pupil size of the horses

**Figure 1:**
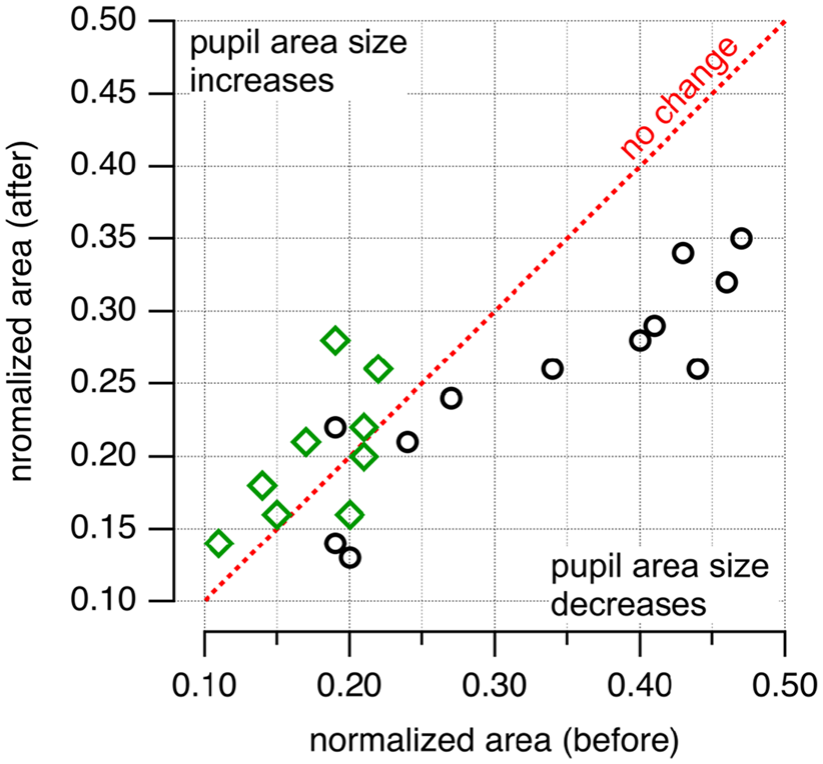
Pupil size changes. Changes in the normalized pupil area are plotted. The black circles show the data from the treated horses, and the green diamonds show the results from the untreated horses. Values on the red broken line indicate that no change in the normalized pupil area occurred; values left to the broken line indicate a decrease in the pupil area size.

The normalized areas of the horses’ eyes in the experimental group before the treatment varied between 0.19 and 0.47, with an average of 0.34 ± 0.11. After the treatment, the pupil size decreased without changes in the ambient light (Table 1, Fig. 1). The normalized area was between 0.13 and 0.35, on average 0.25 ± 0.07 (Table 1, Fig. 1). The differences were statistically significant (paired t-test; n = 12; df = 11; t_statistic_ = 2.19; *P* = 0.042). The effect size was 1.57; the power was 0.999. The values of the normalized areas differed by a factor of 0.58 to 1.17, with an average of 0.78 ± 0.15, indicating a decrease in pupil size after treatment.

#### In control animals, the pupil size did not change

Table 2 and Fig. 1 show the results for the control group of horses placed in the stable lane with a person next to the horse. The horse and the person were allowed to interact. The only difference between treated and control horses was that the control animals did not receive a massage. For the horses in the stable lane, the normalized pupil size was between 0.11 and 0.22, on average 0.18 ± 0.04 after the horses were led into the stable lane and allowed to acclimatize. The pupil size at the end of the session, before the horses were led back to their boxes, was 0.14 and 0.28, on average 0.2 ± 0.04. The differences were not statistically different (paired t-test; n = 10; df = 9; t_statistic_ = − 1.35; *P* = 0.19). The effect size was 0.60; the power was 0.392.

#### The horses’ initial pupil size in the control group was smaller than in the treatment group

At the beginning of the experiment, the normalized areas of the treatment group were larger than the corresponding areas of the control group, 0.34 ± 0.11 versus 0.18 ± 0.04. The difference was statistically significant, t-test; t_statistic_ = − 4.7; *P* = 0.00016; The effect size was 1.93; the power was 0.990.

#### Therapy decreased the pupil size differences between control and treatment groups

After the massage therapy, the horses’ pupil areas were still larger in the experimental group than the corresponding areas of the control group. However, the difference decreased from 0.25 ± 0.07 to 0.20 ± 0.04. The difference was statistically significant, t-test; t_statistic_ = − 2.19; *P* = 0.02; The effect size is 0.88; the power is 0.496.

## Discussion

This work showed that treating horses through massage decreased their pupil diameter. Pupil diameter changes may result from increased ambient brightness or as a mechanism to optimize visual acuity. Furthermore, parasympathetic neurons control the tone of the musculus sphincter pupillae. Hence, activation of the parasympathetic system leads to a decreased pupil size. In contrast, the sympathetically controlled radial muscle of the iris, musculus dilatator pupillae, enlarges the pupil’s diameter. The experimental design was such that luminance was controlled and kept constant for each animal. *The pupil size of most of the horses in the treatment group decreased* after the massage therapy but not in the control group. We concluded that the changes observed originated from elevated parasympathetic and reduced sympathetic activity. The horses relaxed during the equine massage therapy treatment, which the therapist described previously through behavioral observations such as relaxed facial expressions, decreased sensitivity to touch, longer stretches, and relaxed audible exhalations.

The fascination for the eyes, particularly for the pupils, dates back to the 16th Century when the French poet Guillaume de Sallustre described the pupil as “…the window to the soul” ^27^. In the 21st Century, the pupil has gained further interest in science and medicine^9^. While physiological studies were interested in the role of the pupil in regulating the flux of photons to the retina, image formation, and visual acuity^23–25,28–32^, clinicians were interested in the size and area of the pupil during different perceptive, language processing, memory, and decision making, and cognitive challenges^29,33^. In the medical field, pupillometry has also gained importance in diagnostics for anesthesia^9^ or mental stress^14^.

Several previous studies connected the level of stress and pupil diameter^14–16,18,19^. Pedrotti and coworkers^15^ recorded pupil diameter during a simulated driving task. Using a wavelet transform analysis of the data, they successfully indexed the stress manipulation of the driving task. A similar study showed that the pupil diameter during the “Stroop color-word-interference test” could show stress and relaxation^18^.

Stress leads to increased blood cortisol levels. A study in catfish showed that the pupil's diameter correlates with the blood cortisol levels^19^. The authors showed an increased pupil size and heart rate while completing a time-sensitive task. The results indicated an elevated sympathetic activity or stress^14^. Again, the study results agreed with our finding in horses that the smaller pupil area correlates with “relaxed” behavior.

The pupils’ size at the beginning of the treatment suggested that those horses requiring and selected for massage therapy showed a higher stress level. Control horses had, on average, 0.53 times smaller pupil sizes, indicating a more relaxed status when placed in the stable lane. Note that the horses in the control group were selected randomly by the stable owner with no prior assessment of whether treatment would benefit the animal. The control group was to show that standing in the stable lane will not decrease pupil size. The study's results confirmed the expected results and showed a non-significant increase in pupil size. The pupil size of the horses following the treatment almost reached values obtained from horses in the control group; on average, the pupil size of the control animals was 0.8 times smaller than the experimental group after treatment. The changes reflect a change in the balance between parasympathetic and sympathetic activity, emphasizing the parasympathetic activity or relaxation of the horse.

A limitation of the study relates to the frequency with which the pictures were taken. Only two snapshots, one within 5 min before and one within 15 min after the massage, informed on the pupil size. While the averaged data demonstrated an effect, it is not clear how long the change in pupil size lasted. From the snapshots, we could not assess whether certain maneuvers or specific locations resulted in short stress responses during the treatment. Those questions are under examination in a long-term study, where the pupil size of the horse is monitored over extended periods.

## Conclusion

In agreement with previously published results, our study demonstrated that pupillometry constitutes a viable method to monitor the massage effect in horses. The results of this study have shown that equine massage therapy led to a subjective reduction of muscle tension. Those changes were interpreted through stress release during the treatment. The corresponding changes in pupil size were a decrease in diameter and area. They indicated a shift in the balance of the parasympathetic and sympathetic systems’ activity. The changes in the pupil area agreed with the relaxed status of the animals after the therapy. In the next step, we will demonstrate that real-time monitoring is possible, guiding the wellness treatment and optimizing the stress release.

The clinical relevance of the study is the objective measure of the animal’s stress. It provides the therapist a tool to objectively measure the treatment's effect. Further technology development is required until a biofeedback device is available.

## Methods

### Ethics statement

For the study submitted, we took pictures of the horse's eye before and after a massage. The procedure is a non-invasive, observative procedure. The massage treatment was ordered by the horse owner and was completed by a trained horse massage therapist. The animals were healthy horses and did not require the attention of a veterinarian. For the study, nothing in the routine treatment procedure was changed. The image of the animal's eye was added to the routine documentation of the treatment. Retrospectively, the pupil size was measured from the images taken. We obtained informed consent from each horse’s owner to photograph one of the animals' eyes before and after the treatment. All methods are reported in accordance with the ARRIVE guidelines.

### Animal selection, report, and treatment

Our study used eighteen healthy horses in the experimental group and ten in the control group. Six horses in the experimental group were not considered because tracing the pupil’s aperture was impossible. The data in the experimental group reflect the results from the 12 remaining horses. The owners asked an equine sports massage therapist to provide treatment for the animals’ well-being. Before the treatment, the therapist assessed the horse. The animals typically presented with elevated muscle tonus, retraction reflexes on touch, muscle fasciculations or hyper-reflexibility of the muscles involved, and avoidance behavior for specific movements such as canter. Even if only certain muscle groups showed hyper-reflexibility, the massage therapist treated both sides of the horse, following the classical Swedish massage for humans and the equine fascia and trauma release therapy (EFTR®). Liza Kimble has developed the method; it has its roots in fascia massage and trauma-release methods that range from deep, slow pressure to very light touch of the horse. The therapist’s fingers glide in a deep and very slow touch from the caudal toward the cranial section of the horse, against the fur’s direction. The fingers follow parallel to the spine (about 2–3 cm away) and do not lose contact during this procedure. This method continues with the treatment of the ventral section of the horse. The therapist lightly touches the horse’s belly with the palm and lets the hand glide over the fur in soft circles. For geldings, the castration scars are treated carefully to loosen. This is important because tight castration scars can negatively affect the adductors and activation of the hindlegs’ motion. After the EFTR®, the therapist uses classical Swedish massage techniques, similar to human massage therapy.

The therapist also applied patterns of vibration delivered by a handheld device (Atlasbalans, Sollentuna, Sweden) to the tensed muscle group(s). The treatment took up to an hour, after which the horses appeared relaxed and no longer showed the initial muscle tension, sensitivity to touch, and stretching. Note that no conditions that required veterinary attention were treated.

The control group included ten horses. The horse owners selected the horses randomly. The only criterion for the horse owner was that they should not present with elevated muscle tonus, retraction reflexes on touch, muscle fasciculations or hyper-reflexibility of the muscles involved, and avoidance behavior for specific movements such as canter. The ten horses in the control group stood approximately the same time in the stable lane a treatment would take, with a person next to the horse. The horse and the person were allowed to interact, and interactions with passers were permitted, except for the massage of the animal.

The control horses were for dressage; the experimental group comprised jumping, dressage, eventing, and leisure horses; one horse was partially retired. The data were collected from horses living in five different stables.

### Image capture

**Figure 2:**
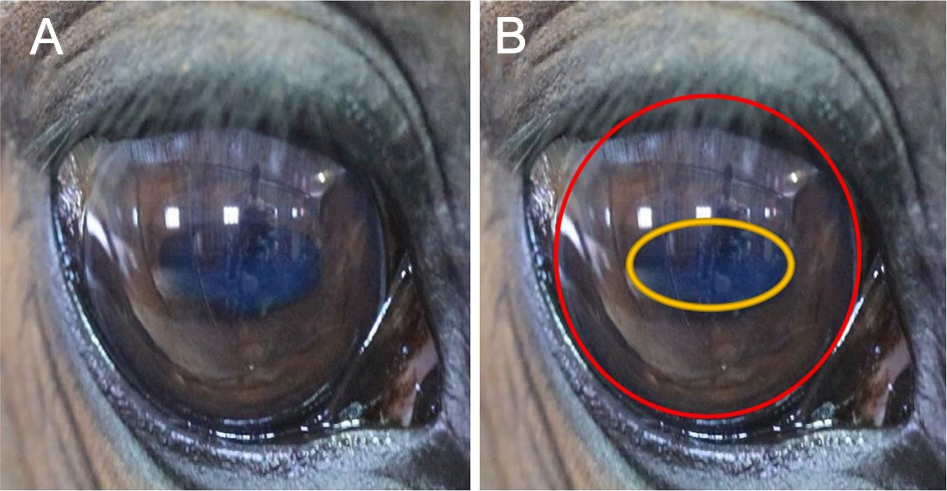
Pupil size measurements. (**A**) shows the image of one eye of the horse. The outline of the iris is the white line in (**A**), traced with the line tool as a red line in (**B**)**.** The number of pixels in this area provides the reference values, as they will not change over time. The pupil is the oval darker area in (**A**)**,** traced with an orange line in (**B**). The number of pixels in this area defines the pupil area's size, which changes over time. To study whether the size of the pupil changed, the ratio of the pixels in the pupil and the reference area is calculated and compared before and after the treatment.

The horse was removed from its box for treatment and tied in the stable lane. Before starting the therapy, we took five to ten images of the horse’s eye (Fig. 2A) with an iPhone-XS camera. After the treatment, we photographed the same eye under the same illumination (Fig. 2B).

Another important factor related to the time a horse received its massage. The pupil size is modulated by cortisol levels, which change over time. We considered this a confounding factor, and all treatments were done within six hours between 13:00 and 19:00.

### Image analysis

To quantify the treatment effect, pupil areas were compared before and after the treatment. Images were transferred to a laptop computer and processed with Fiji (ImageJ). Each photo of the horse head was opened to zoom into the region of interest, the eye. The identifiable landmarks for the area measurements, the line identifying the eye bulb's sclera, and the pupil (Fig. 2) were traced with the elliptical or brush selection tool in ImageJ. The number of pixels for each area was determined with the measuring tool. The ratio between the number of pixels in the pupil and reference areas was calculated for the condition before and after the treatment and in the control group for the starting and ending time for the horse in the stable lane. The change in the ratio was then reported for each of the animals. Values below 1 referred to a decrease in pupil size, while values above 1 indicated an increase.

### Statistical analysis

Averages and standard deviations were calculated for the ratios of the pupil-to-iris area. For the experimental group, it was before and after the treatment, and for the control group, before and after the horse was placed in the stable lane. The normalized size of the pupil was compared with a two-tailed paired t-test within groups and a two-tailed t-test between groups. Statistical significance was established for an alpha of 0.05. Effect size and power calculations are completed using G*Power 3.1^34^.

## Data Availability

All data analyzed have been shown in the paper. The raw data are available upon request from the corresponding author.
